# Identification of an immune subtype-related prognostic signature of clear cell renal cell carcinoma based on single-cell sequencing analysis

**DOI:** 10.3389/fonc.2023.1067987

**Published:** 2023-03-22

**Authors:** Zongyao Fan, Hewei Xu, Qingyu Ge, Weilong Li, Junjie Zhang, Yannan Pu, Zhengsen Chen, Sicong Zhang, Jun Xue, Baixin Shen, Liucheng Ding, Zhongqing Wei

**Affiliations:** ^1^ Department of Urology, The Second Affiliated Hospital of Nanjing Medical University, Nanjing, China; ^2^ Department of Urology, The Second Clinical Medical College of Nanjing Medical University, Nanjing, China; ^3^ Department of Rehabilitation Medicine, The Second Affiliated Hospital of Nanjing Medical University, Nanjing, China

**Keywords:** ccRCC, single-cell sequencing analysis, immune, prognostic signature, vmp1

## Abstract

**Background:**

There is growing evidence that immune cells are strongly associated with the prognosis and treatment of clear cell renal cell carcinoma (ccRCC). Our aim is to construct an immune subtype-related model to predict the prognosis of ccRCC patients and to provide guidance for finding appropriate treatment strategies.

**Methods:**

Based on single-cell analysis of the GSE152938 dataset from the GEO database, we defined the immune subtype-related genes in ccRCC. Immediately afterwards, we used Cox regression and Lasso regression to build a prognostic model based on TCGA database. Then, we carried out a series of evaluation analyses around the model. Finally, we proved the role of VMP1 in ccRCC by cellular assays.

**Result:**

Initially, based on TCGA ccRCC patient data and GEO ccRCC single-cell data, we successfully constructed a prognostic model consisting of five genes. Survival analysis showed that the higher the risk score, the worse the prognosis. We also found that the model had high predictive accuracy for patient prognosis through ROC analysis. In addition, we found that patients in the high-risk group had stronger immune cell infiltration and higher levels of immune checkpoint gene expression. Finally, cellular experiments demonstrated that when the VMP1 gene was knocked down, 786-O cells showed reduced proliferation, migration, and invasion ability and increased levels of apoptosis.

**Conclusion:**

Our study can provide a reference for the diagnosis and treatment of patients with ccRCC.

## 1 Introduction

Renal cell carcinoma (RCC) is one of the most common and deadly malignancies of the urinary tract, with an annual morbidity rate of 2.2% and a mortality rate of 1.8% (1). Clear cell renal cell carcinoma (ccRCC) is the most common histological type of RCC, making up about 80% of all cases (2, 3). Currently, the preferred clinical treatment is partial or radical nephrectomy for patients with stage I or II renal cell carcinoma (4). However, about 30% of patients have metastasized at first diagnosis and the 5 years survival rate for this group of patients is low because ccRCC is not sensitive to radiotherapy and chemotherapy (5, 6). Therefore, it is important to find new therapeutic tactics to improve the prognosis of ccRCC.

The tumor microenvironment (TME) is a complex dynamic multicellular ecosystem consisting of a variety of components such as immune cells, stromal cells, cancer cells, neuronal cells, blood vessels, and various growth factors (7, 8). Immune cells in the TME have been considered a key and central area of oncology research, playing a valuable role in the prognosis of malignancies, and in treatment resistance (9). Obradovic et al. demonstrated that TREM2/APOE/C1Q-positive macrophage infiltration is a potential prognostic biomarker for ccRCC recurrence, as well as a candidate therapeutic target (10), and Errarte et al. proved the implication of CAF (cancer-associated fibroblasts) in the proliferation, angiogenesis, metastasis development and drug resistance during RCC tumourigenesis. This fact assumes that CAF is a potential clinical tool for the diagnosis, prognosis and treatment of ccRCC (11). Furthermore, TME-related biomarkers were found to predict prognosis for ccRCC patients as novel targets for immunotherapy (12). In recent years, various immunotherapeutic strategies, comprising anti-PD-1, anti-PD-L1 and anti-CTLA-4, are recommended as the mainstay of treatment for advanced RCC. However, the majority of patients who receive immunotherapy experience primary and acquired drug resistance, which ultimately causes treatment failure (13, 14). Therefore, the discovery of new targets for immunotherapy is of great importance.

Single-cell RNA sequencing (SCQ) is used to study cell heterogeneity and to identify different cell types within heterogeneous cell populations. Unlike traditional RNA sequencing, SCQ will help to understand the differences between different cells at the gene and gene expression levels during disease progression (15, 16). SCQ is now widely used in the study of various diseases, and results have been achieved (17, 18).

In our study, we first performed dimensionality reduction, clustering and cell type annotation analysis on the SCQ data of ccRCC. Through these analyses, we classified the different tumor cells as immune and non-immune components and successfully obtained marker genes for cells in the immune group. A prognostic model for ccRCC patients was constructed based on these genes and clinical information and transcriptome sequencing of ccRCC patients from the The Cancer Genome Atlas (TCGA) database. This model precisely assesses the prognosis of ccRCC patients and is associated with the immune microenvironment. Finally, we validated the role of VMP1, the important gene in the model, through cellular experiments. Our study offers novel ideas for the diagnosis and treatment of ccRCC.

## 2 Methods

**Figure 1:**
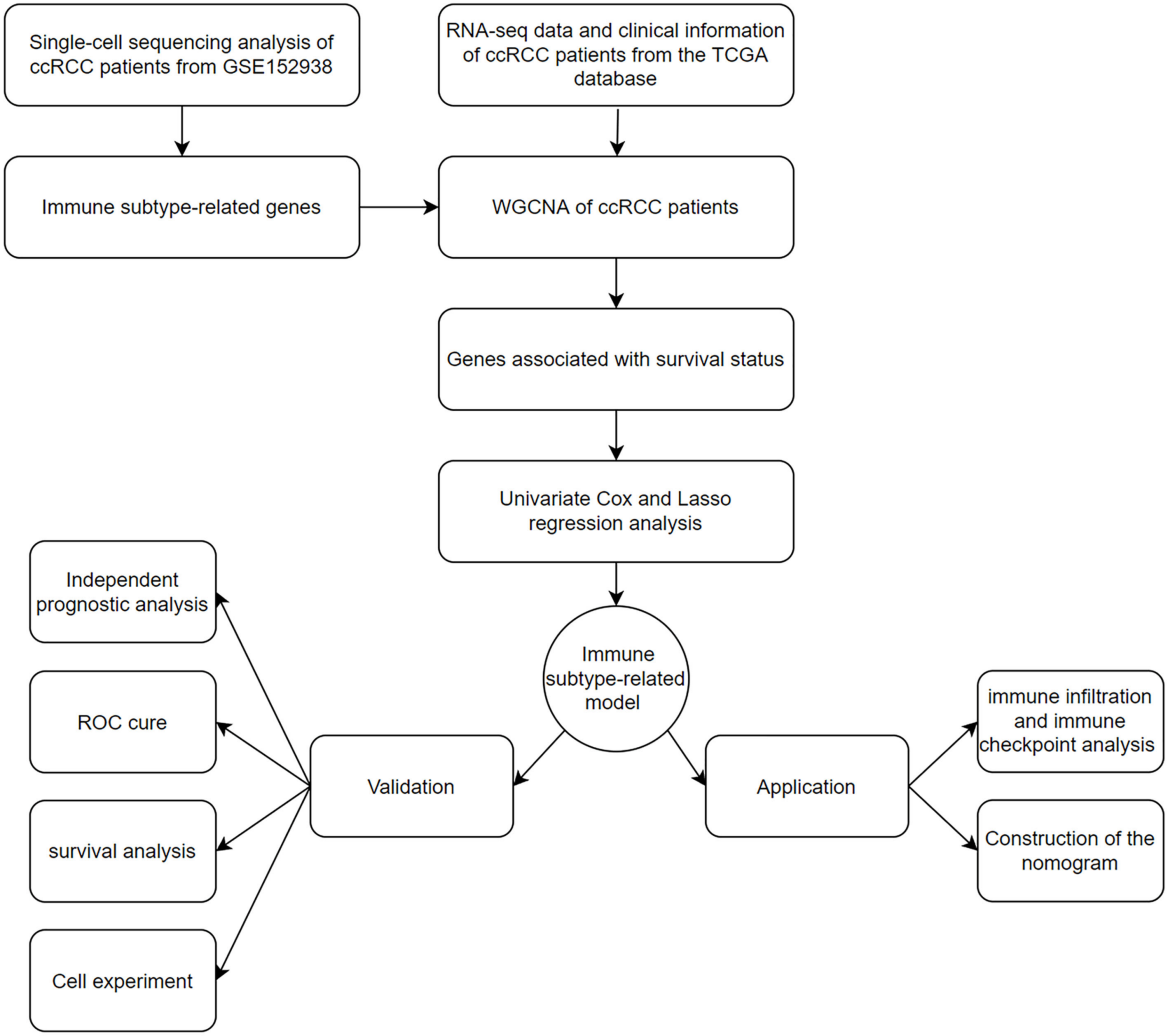
The flowchart of data collection and analysis in this study.

A flowchart of our work was shown in **Figure 1**.

### 2.1 Data source and preprocessing

The ccRCC SCQ dataset GSE152938 was downloaded from the GEO database, including 1 normal kidney sample, 2 ccRCC samples, 1 chromophobe renal cell carcinoma sample and 1 papillary renal cell carcinoma sample. Because this article was designed to study the prognosis of patients with ccRCC, we removed other types of samples. Next, we perform quality control on the SCQ data, we selected genes expressed in at least three cells and cells with total gene expression between 300 and 3000 for the next analysis. And, cells with mitochondrial gene expression greater than 5% of total gene expression were also excluded. The transcriptome RNA-seq data and its corresponding clinical information were acquired from the TCGA database, comprising 539 ccRCC data and 72 normal data. And, to ensure the accuracy of the study, 530 ccRCC samples that included complete clinical information were selected for further analysis.

### 2.2 SCQ data analysis

First, we normalized the ccRCC SCQ data filtered in the previous step by the method of “LogNormalize”. Then, due to the sheer volume of cells, we classified them by the marker genes expressed by each cell, and merged the similar categories, through the method of principal component analysis (PCA) dimension reduction. Finally, with the help of the function of “SingleR”, we annotated cell types according to their marker genes, and we used the “FindAllMarkers” function to obtain marker genes for different cell types.

### 2.3 Weighted gene co-expression network analysis

WGCNA is a systematic statistical approach that can group genes with analogous expression patterns and illustrate the relationship between genes of a particular group and specific traits (19). In our study, we used this method to obtain the set of genes associated with clinical traits. First, we performed an initial screening of the samples, excluding non-renal cancer patients and genes with small fluctuations. Then, to improve the accuracy of the screening, we transformed the adjacency matrix into a topological overlap matrix (TOM) and set the minimum group size to 30. Finally, we merge similar groups and output the resulting graph and data.

### 2.4 Construction of the immune subtype-related prognostic model

Firstly, we combined the above-obtained genes with TCGA transcriptome data to obtain the immune subtype-related gene expression data. Subsequently, we merged the expression data with their survival status and performed a univariate Cox analysis to identify genes associated with prognosis. Then, the genes were further selected by the way of Lasso regression analysis, and through this we can get the model genes. Finally, we calculated the risk score for each ccRCC patient based on the formula and with the help of the median risk score, we were able to classify the patients into two risk groups.

### 2.5 Evaluation of the model

We analyzed whether the risk score was an independent prognostic factor by the measure of Cox analysis. Then, we assessed the predictive effect of the model by plotting the survival curves of the ccRCC patients, and we make the most of the ROC curve to evaluate the accuracy and sensitivity of this model. In the last, we plot the patient’s survival status on an axis with the risk score as the horizontal coordinate to give a better visualisation of each patient’s survival status.

### 2.6 Analysis of immune function

With the help of the results of 7 kinds of immune infiltration in ccRCC downloaded from the TIMER database, we showed the difference in the level of immune infiltration between ccRCC patients in two groups. Meanwhile, we also investigated the difference in the expression level of immune checkpoint-related genes between ccRCC patients in two groups.

### 2.7 Construction of the nomogram

In our study, by combining each patient’s risk score of our model with clinical information, we successfully constructed a nomogram that can predict the patient’s risk of death. Then we used ROC to assess the accuracy of nomogram in predicting patient outcomes

### 2.8 Cell culture and transfection

CcRCC cell-lines 786-O was purchased from the Chinese Academy of Sciences Committee on Type Culture Collection Cell Bank (Shanghai, China) and were cultured in RPMI-1640 medium (Gibco, USA) supplemented with 10% FBS (Gibco, USA). siRNA VMP1 and siRNA negative control were purchased from RiboBio (Guangzhou, China), and transfected with Lipofectamine 2000 reagent (Invitrogen, CA, USA).

### 2.9 RNA extraction and quantitative real-time polymerase chain reaction

Total RNAs of 786-O were extracted using the TRIzol reagent. Then, we made use of a reverse transcription kit from (vazyme, China) to obtain cDNA. We detected the relative expression level of the target gene by the measure of qRT-PCR based on the 2-ΔΔCt method.

### 2.10 Cell proliferation analysis

5-ethynyl-29-deoxyuridine (EdU) assay was performed based on the manufacturer’s instructions (RiboBio, Guangzhou, China). The 786-O cells were first inoculated in 24-well plates, followed by incubation with EdU reagent for 2h. Finally, after labelling the DNA with 2-(4-Amidinophenyl)-6-indolecarbamidine dihydrochloride (DAPI), the cell images were inspected under a fluorescent microscope.

### 2.11 Transwell assay

To assess the migratory capacity of the cells, we adjusted the 24-well plates (Nset, China) by transwell culture chambers (Corning, USA). Cells were inoculated into 200μL of the medium in the upper chambers without serum. The lower layer of the chamber is 700μL of medium containing 10%FBS. For cell invasion ability, pre-lay a layer of Matrigel over the chambers, the rest of the steps are the same as above. After 24h incubation in the cell incubator, the medium was discarded and the cells were wiped from the inside of the bottom of the chambers using a cotton swab. Finally, after fixing them with methanol and staining the cells at the bottom of the chamber with crystal violet, images of the cells were taken using a microscope.

### 2.12 Scratch wound healing assay

Cells in logarithmic growth phase were inoculated in 6-well plates. When the cells reached about 90%, 200μL tips were used to draw 2 vertical lines along the vertical direction, and after washing out the cell debris, the complete medium was replaced with a medium containing 1% FBS. After 24 hours, the distance the cells migrated to the scratched area was carefully observed under the microscope and this was used to test the migration ability of the cells.

### 2.13 Apoptosis detection using flow cytometry

Cells were first digested with trypsin and washed twice with PBS. Then, according to the Annexin V-FITC/PI Apoptosis Detection Kit (vazyme, China) guidelines, cells were incubated with Annexin V-FITC and PI in a dark environment for 10 minutes. Finally, the rate of apoptosis was measured using flow cytometry.

### 2.14 Statistical analysis

Bioinformatics analysis was conducted using the R software (V. 4.1.2). The quantification and graphing of the experiment data was conducted using Image J software (V.1.8.0) and GraphPad Prism (V.9.0). All measurement data are shown as the mean ± SD. The data differences between the two groups were analyzed by Student’s t-test and P-values less than 0.05 were considered significant in all tests.

## 3 Results

### 3.1 SCQ data analysis and identification of immune-related genes

**Figure 2:**
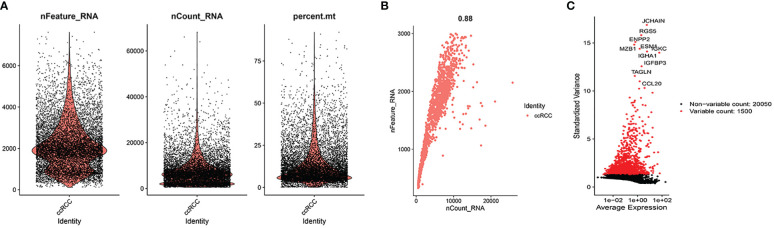
Quality control. **(A)** When gene expression levels in cells of ccRCC samples were in the range of 300-3000, the distribution of each cell was relatively even. At the same time, we also found the mitochondrial genes of the majority of cells were <5%. **(B)** The cells were uniformly distributed in the ccRCC samples and the gene expression levels were positively associated with the amount of gene expression (0.88). **(C)** 10 hypervariable genes.

As illustrated in **Figure 2A**, we found a relatively even distribution of cells with gene expression levels between 300 and 3000, and the mitochondrial genes of the majority of cells were <5%. Using the above criteria to screen the cells, we successfully obtained 1759 cells. **Figure 2B** showed that these cells were uniformly distributed in the ccRCC samples and that the gene expression levels were positively associated with the amount of gene expression (0.88). This indicated that the screened cells are suitable for further analysis. **Figure 2C** showed the ten most variable genes in selected cells, including JCHAIN, RGS5, ENPP2 and MZB1.

**Figure 3:**
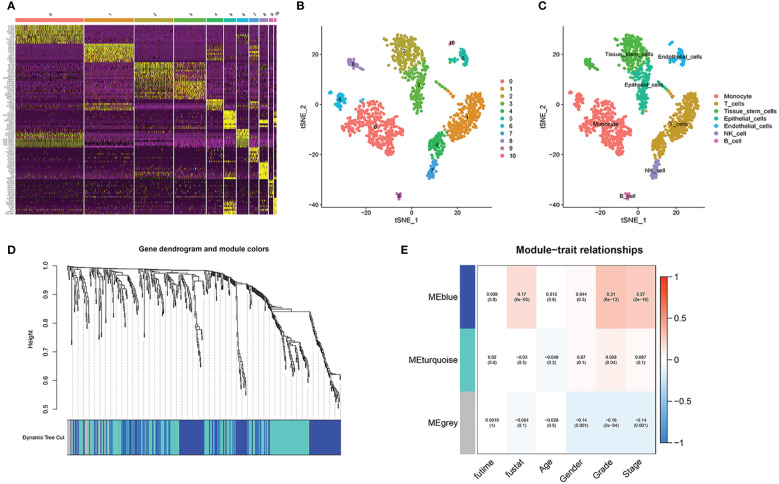
Single-cell sequencing analysis. **(A)** 10 highest expressed genes in each cluster. **(B, C)** the distribution and annotations of these 11 clusters. **(D, E)** WGCNA found that MEblue was closely related to the score of survival status.

After PCA descending treatment, these cells were divided into 11 clusters. In **Figure 3A**, we could find 10 highest expressed genes in each cluster. In **Figure 3B**, we could find the distribution of these 11 clusters. With the help of the function of “SingleR”, we annotated cell types according to their marker genes, and the clusters associated with immune cells are 0, 1, 4, 6, 7, 9 (**Figure 3C**). We then used the “FindAllMarkers” function to acquire 858 immune subtype-related genes.

### 3.2 Weighted gene co-expression network analysis

In TCGA cohort, with the help of WGCNA, we got the gene modules related to the patient’s survival status. By using a soft threshold of 4 and a minimum module gene count of 30, we succeeded in obtaining 3 modules related to clinical traits (**Figures 3D, E**). Because we wanted to analyze patients’ prognosis, we selected genes associated with the patients’ survival status for further analysis.

### 3.3 Construction of the immune subtype-related prognostic model

**Figure 4:**
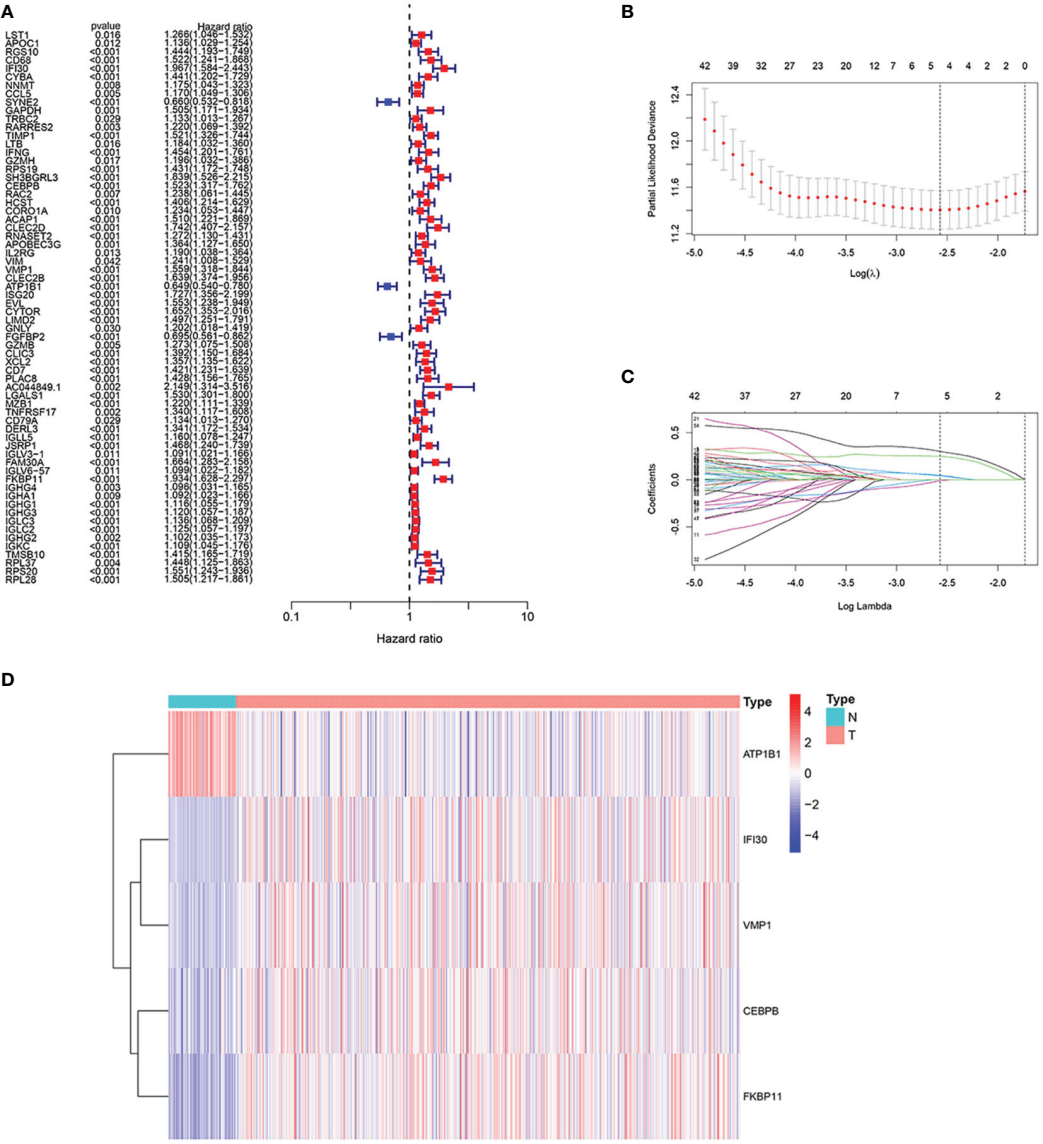
Construction of the prognostic model. **(A)** We succeeded in obtaining 66 genes associated with the prognosis of the patients through the univariate Cox analysis, 63 of which had a hazard ratio (HR) > 1. **(B, C)** 5 genes were selected to construct the prognostic model by Lasso regression. **(D)** Expression of 5 model genes in the transcriptome sequencing of normal and ccRCC patients.

First, a differential analysis was performed based on genes obtained in the previous step in the TCGA cohort to obtain the differential genes in the tumor and normal groups. Then, as shown in **Figure 4A**, we succeeded in obtaining 66 genes related to the prognosis of ccRCC patients through univariate Cox analysis, 63 of which had a hazard ratio (HR) > 1. In the last, after randomizing patients into the training and validation set, we carried out Lasso regression analysis on these 64 genes, and the result showed when the number of genes included is 5, the gene contraction tended to be stabilized and the partial likelihood deviation was minimized (**Figures 4B, C**). We finally obtained 5 model genes, including IFI30, CEBPB, VMP1, ATP1B1, and FKBP11, and we found that gene ATP1B1 was highly expressed in normal patients, while IFI30, CEBPB, VMP1, and FKBP11 were highly expressed in ccRCC patients (**Figure 4D**). The names and the coefficients of the prognostic genes were listed in **Table 1**. Risk score = IFI30*0.252 + CEBPB *0.050 + VMP1*0.041 + ATP1B1*(-0.007) + FKBP11*0.302. We then used median patient risk values to classify patients into two groups for further analysis.

**Table 1:** Genes used for model building and their Coefficients.

Gene	Coefficients
IFI30	0.251764
CEBPB	0.049804
VMP1	0.041372
ATP1B1	-0.007404
FKBP11	0.301602

Genes and their coefficients used to construct prognostic models. The risk score = IFI30*0.251764 + CEBPB *0.049804 + VMP1*0.041372 + ATP1B1*(-0.007404) + FKBP11*0.301602.

### 3.4 Validation of the immune subtype-related prognostic model

**Figure 5:**
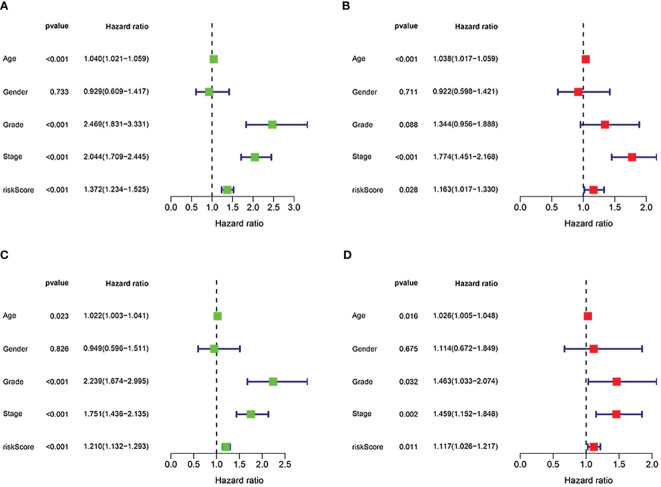
Independent prognostic analysis of the signature. **(A, B)** Cox regression revealed that the risk score was an independent prognostic factor in ccRCC patients in training group. **(C, D)** Cox regression revealed that the risk score was an independent prognostic factor in ccRCC patients in validation group.

**Figure 6:**
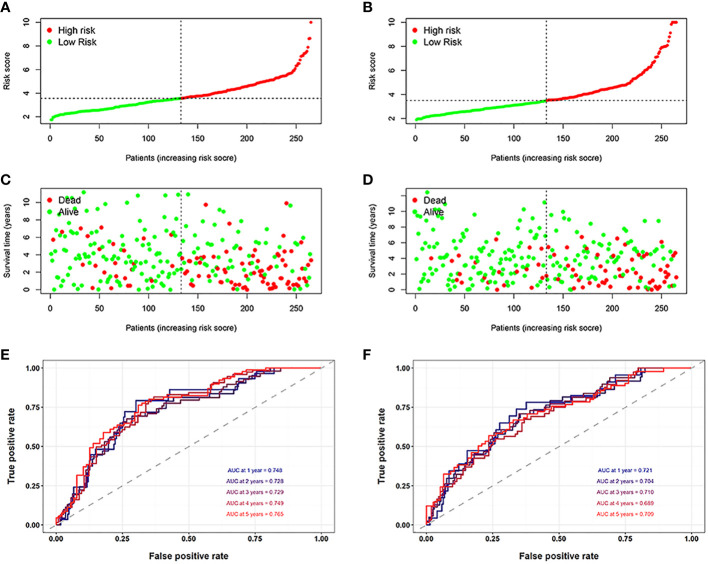
Assessment of the model. **(A, B)** The distribution of patients’ risk scores in the training and validation groups. **(C, D)** With increasing risk scores, the chance of patient death increased. **(E, F)** The ROC curves for 1, 2, 3, 4 and 5 years for both datasets.

**Figure 7:**
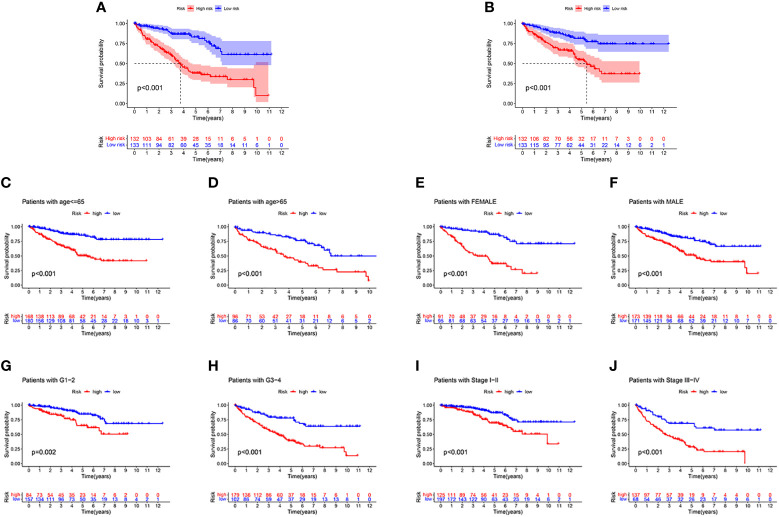
The survival analysis. **(A, B)** The survival analysis between high-risk groups and low risk groups between the training cohort and the validation cohort. **(C–J)** A more rapid decline in survival in the high-risk group than in the low-risk group, irrespective of age, gender grade and stage.

First, to explore whether risk scores were an independent factor of influence for ccRCC patients, we performed univariate and multivariate Cox regression on age, gender, grade, stage and risk score in ccRCC patients. The presentation of the results showed that in both the training and validation sets, the risk score was an independent prognostic factor (**Figure 5**). We then examined the relationship between patients’ risk scores and survival status. In **Figures 6A, B**, we could find the distribution of patients’ risk scores in ccRCC patients. And, with increasing risk scores, the chance of patient death increased (**Figures 6C, D**). Next, to validate the accuracy of the model, we plotted ROC curves for 1, 2, 3, 4 and 5 years for both datasets. We found the area under the curve (AUC) was found to be almost greater than 0.7 for both datasets from 1 to 5 years, suggesting that the model had good stability and accuracy in predicting patient prognosis (**Figures 6E, F**). Finally, to further test the credibility of the model, we performed a survival analysis in the ccRCC patients (**Figures 7A, B**). At the same time, a further, more specific categorical survival analysis was carried out for all ccRCC patients. The results showed a more rapid decline in survival of ccRCC patients in the high-risk group, irrespective of age, gender grade and stage (**Figures 7C–J**).

### 3.5 Evaluation of immune infiltration and immune checkpoint

**Figure 8:**
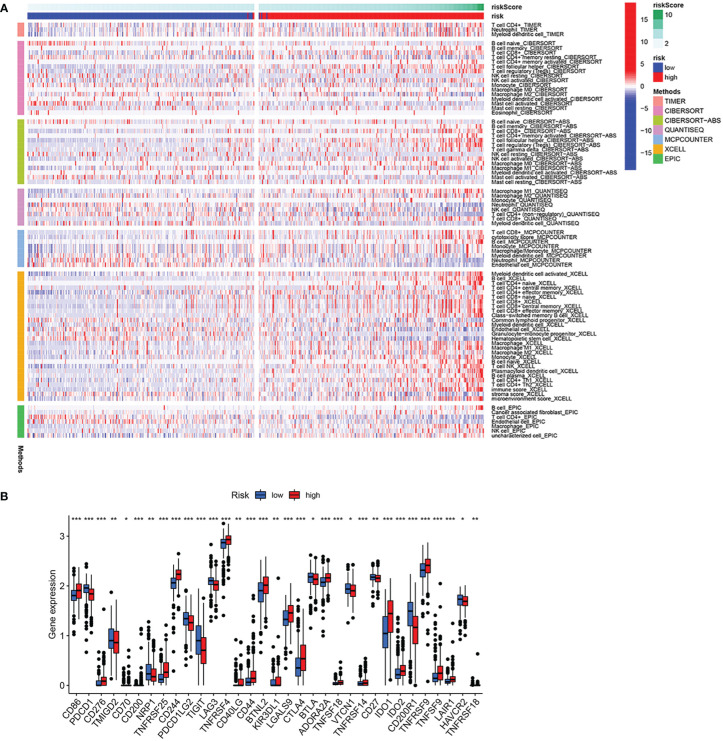
Analysis of immune infiltration and immune checkpoint. **(A)** Heatmap of immune cell infiltration in high-risk group and low-risk group. **(B)** Differential expression of immune checkpoint genes in high-risk group and low-risk group. *p<0.05, **p<0.01, ***p<0.001.

As shown in the above analysis, patients in the high-risk group had significantly poorer survival. We therefore wanted to investigate whether there were differences in immune function in order to guide the treatment of the disease in some sense. The results showed more immune cell infiltration in the high-risk group, consisting of T cells, B cells and macrophage cells (**Figure 8A**). Furthermore, almost all immune checkpoint genes were also more highly expressed in the high-risk group (**Figure 8B**), indicating that it is possible that high-risk group ccRCC patients may receive more benefit from immunotherapy.

### 3.6 Construction of the nomogram

**Figure 9:**
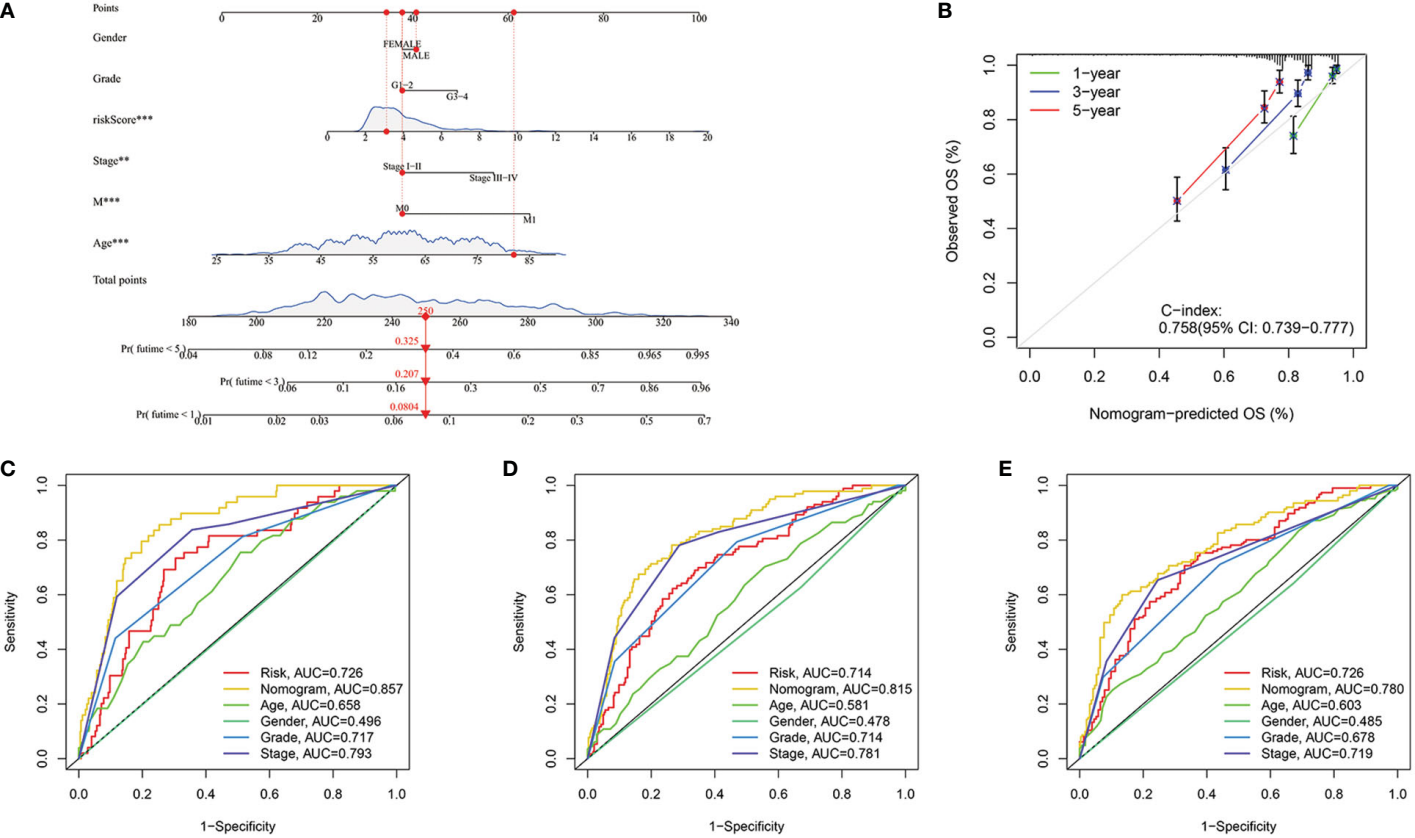
Construction of the nomogram. **(A)** Nomogram to predict the probability of mortality at 1, 3, and 5 years. **(B)** The C-index of the nomogram. **(C–E)** ROC curve of the nomogram in 1, 3 and 5 years were 0.857, 0.815 and 0.780 respectively. **p<0.01, ***p<0.001.

In order to better predict the prognosis of ccRCC patients, a nomogram was constructed including clinical information and risk score. In **Figure 9A**, with the use of gender, age, total stage, M stage, grade and risk score values for the patient “TCGA-CZ-4853”, we predicted his mortality rates of 0.0804, 0.207 and 0.325 at 1, 3 and 5 years. Next, we constructed a calibration curve (**Figure 9B**) and found that the nomogram was a good predictor of prognosis at 1, 3 and 5 years for ccRCC patients. In addition, ROC analysis was carried out to better assess the accuracy of the nomogram. The results showed that both the 1 year, 3 year, and 5 years, nomogram was more accurate than clinical information (**Figures 9C–E**).

### 3.7 Effect of VMP1 knockdown on the proliferation of ccRCC cells

**Figure 10:**
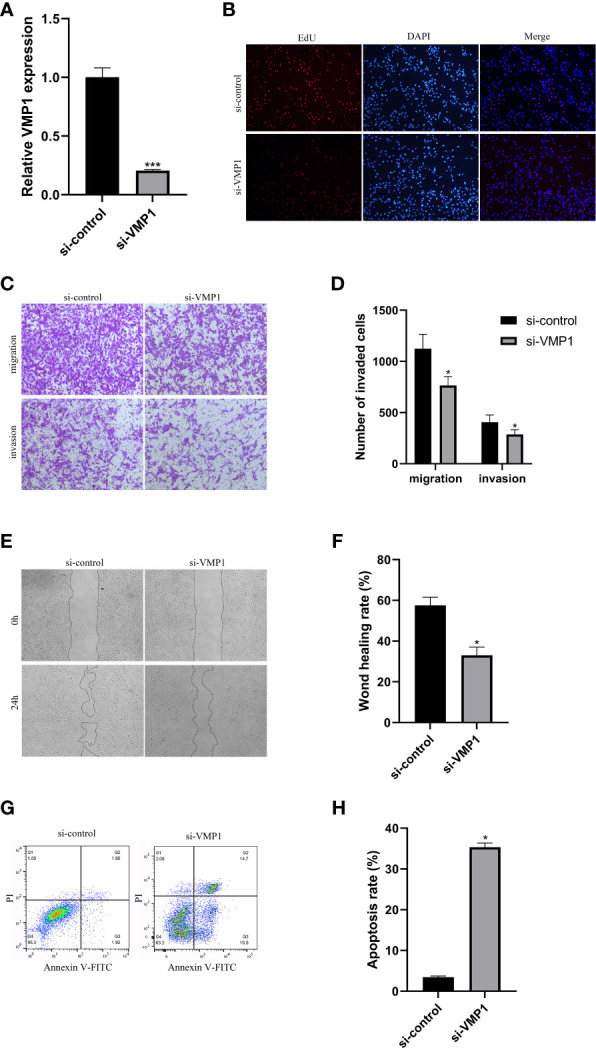
Cell experiments. **(A)** qRT-PCR analysis was performed to confirm the knockdown of gene VMP1. **(B)** EdU assay suggested that the proliferation ability of 786-O cells was reduced with VMP1 knockdown. **(C, D)** Transwell assay showed that the migration and invasion of 786-O cells were reduced with VMP1 knockdown. **(E, F)** Scratch assay showed that the migration of 786-O cells was reduced with VMP1 knockdown. **(G, H)** Cell apoptosis assay showed that the apoptosis rate of 786-O cells was Increased with VMP1 knockdown. *p<0.05, ***p<0.001.

To assess the knockdown efficiency of the VMP1 in 786-O cells, we examined the expression of the VMP1 in the 786-O cell line by qRT-PCR. **Figure 10A** showed significant downregulation of VMP1 expression levels in the 786-O cells after siRNA transfection, suggesting that further studies are feasible and meaningful. The EdU assay was used to test whether VMP1 knockdown had an effect on the proliferation of 786-O cells, which showed that the proliferation of cells was suppressed after the vmp1 gene was knocked down (**Figure 10B**).

### 3.8 Effect of VMP1 knockdown on the migration and invasion of ccRCC cells

The effect of decreased VMP1 expression on cell migration and invasion was examined by the transwell method. Results displayed that the decreased expression of VMP1 also impaired cell migration and invasion (**Figures 10C, D**). Scratch healing assays also showed a significantly slower wound healing rate in 786-O cells with a decreased expression of the VMP1 (**Figures 10E, F**).

### 3.9 Effect of VMP1 knockdown on the apoptosis of ccRCC cells

We analyzed the influence of VMP1 on the apoptosis of 786-O cells. The results indicated that the apoptosis level in the low VMP1 expression group was significantly higher compared to NC group (**Figures 10G, H**).

## 4 Discussion

As the most common and malignant subtype of RCC, the main treatment options for advanced ccRCC consist of palliative tumor resection, targeted therapy and immunotherapy due to its insensitivity to radiotherapy and chemotherapy (20). Although a large number of ccRCC patients currently have improved overall survival rates as a result of immunotherapy, there are still some patients who have poor outcomes (21). These suggest that our understanding of the immune microenvironment of ccRCC is far from adequate and we need to continue to explore its mechanisms and find new prognostic markers and therapeutic targets.

In this study, we analyzed SCQ data from ccRCC to classify cells into immune and non-immune groups and extracted marker genes from the immune group. We then performed Cox and Lasson regression analyses based on these marker genes and constructed an immune subtype-related prognostic model. Each patient was then divided into two groups by calculating risk scores, and the model was found to be an accurate predictor of patient prognosis through survival analysis, AUC and other analyses. We next found higher levels of immune infiltration and immune checkpoint genes expression in the high-risk group, indicating that patients in the high-risk group are able to receive more benefits from immunotherapy. Finally, our cellular experiments displayed that the proliferation and migration of kidney cancer cells were reduced and apoptosis levels were increased after vacuole membrane protein 1 (VMP1) knockdown, revealing that it may be a key oncogene and a possible breakthrough point for treatment.

Our risk model includes 5 genes, all of which take part in the regulation of cancer. Interferon γ-inducible protein 30 (IFI30) is a reductase localized in lysosomes and expressed mainly in antigen-presenting cells, including B cells, T cells and macrophages, that catalyzes the reduction of disulfide bonds (22, 23). IFI30 can promote breast cancer proliferation, migration and invasion through cellular autophagy, and promote melanoma development by modulating tolerance to autoantigens (23, 24). CCAAT/enhancer-binding protein B (CEBPB)is a member of the family of transcription factors of the basic-leucine zipper class. When subjected to external stimuli, its expression can be increased, promoting the expression of downstream inflammatory factors and thus promoting the proliferation and migration of glioblastoma cells (25). FK506 binding protein 11 (FKBP11) has been reported to be highly expressed in melanoma, hepatocellular carcinoma and oral cancer and to promote the development of oral cancer by regulating the cell cycle and apoptosis through the P53 pathway (26–28). As ccRCC progresses, increased methylation of the promoter of ATPase Na/K transporting subunit beta 1 (ATP1B1) decreases its expression in cancer, thereby inhibiting tumor progression and acting as a cancer suppressor (29, 30). VMP1, previously thought to be a pancreatitis-associated protein (31), has recently been demonstrated to promote glioma development and Kras-mediated pancreatic cancer initiation by regulating cellular autophagy (32, 33). In addition, in acute myeloid leukemia, HER2 positive breast cancer and ovarian cancer, the poor prognosis of patients is strongly associated with high expression of VMP1 (34–36). However, overexpression of VMP1 inhibited the metastasis, proliferation and increased their sensitivity to chemotherapeutic drug, 5-fluorouracil, in colorectal cancer cells (37). There are no similar studies in ccRCC patients, so this paper focused on VMP1. We found that poor prognosis in ccRCC patients was related to high VMP1 expression and that knockdown of VMP1 inhibited cell growth and induced apoptosis.

CcRCC is one of the most immunologically infiltrative tumors of the urinary tract and immunotherapy is the main treatment option for advanced kidney cancer (38). Therefore, it is important to know the immune function of each patient to control the progression of the tumor and prolong the prognosis of the patient and to look for new prognostic markers to extend the survival time of patients. We propose a new model for immune subtypes with the help of SCQ analysis of ccRCC. Patients in the high-risk group have higher levels of immune infiltration, which has implications for guiding treatment.

In summary, we have developed a new prognostic model based on the results of single-cell analysis, which can accurately predict the survival time of ccRCC patients and has implications for guiding immunotherapy. We have initially validated the effect of VMP1 on ccRCC cell function, and we will further explore the specific mechanisms of VMP1 at the cellular level to provide new targets for the diagnosis and treatment of ccRCC.

## 5 Conclusions

We constructed an immune subtype-related prognostic signature of ccRCC, and demonstrated the role of VMP1 in ccRCC by cellular assays. These can accurately assess the prognosis of patients with ccRCC and provide a new target for treatment.

## Data availability statement

The original contributions presented in the study are included in the article/supplementary material. Further inquiries can be directed to the corresponding author.

## Author contributions

ZF, HX and QG were responsible for the design of this study. WL, JZ, YP, ZC, SZ and JX were involved in database search and statistical analyses. ZF, HX, QG, LD and BS were involved in the writing of manuscript. ZW was responsible for the submission of the final version of the paper. All authors contributed to the article and approved the submitted version.
